# Research on Bamboo Scrimber’s Compressive Creep Behaviour Based on Different Kelvin-Voigt Models

**DOI:** 10.3390/ma19061226

**Published:** 2026-03-20

**Authors:** Zhiwei Miao, Songsong Sun, Jiahong Fu, Xiaolin Gong, Weiwei Wang, Xiaomei Xu

**Affiliations:** 1 College of Automobile and Traffic Engineering, Nanjing Forestry University, Nanjing 210037, China; mzw@njfu.edu.cn (Z.M.); sunsong1987@126.com (S.S.); xiaolin_gong@njfu.edu.cn (X.G.); wangweiwei@njfu.edu.cn (W.W.); xxm120480@126.com (X.X.); 2 College of Engineering, Hangzhou City University, Hangzhou 310015, China

**Keywords:** bamboo material, mechanical property, Caputo model, variable-order fractional derivative, viscoelastic mechanics

## Abstract

Creep is one of the most important factors that should be considered during the application of composite materials in modern industry. In this work, bamboo scrimber, a commonly used natural fibre-reinforced composite material manufactured via hot pressing, was investigated to determine its creep property under compressive loading. Its creep evolution history alongside time-varying load history were analysed. In addition, variations of the Kelvin-Voigt model were used to analyse the mechanical constitutive relation of the material. The key finding of this paper is that the creep strain growth behaviour of bamboo crimper mostly depends on the stress level acting on it. Moreover, the VOF (variable-order Caputo fractional) derivative-based Kelvin-Voigt model is more suitable than the traditional model, as it simulates the dynamics of the time–strain relationship of bamboo scrimber at all relevant stress levels. The effect of stress level on the main model parameters was also analysed through detailed function models. These benefits suggest that the proposed model is significantly useful in terms of informing the design and implementation of bamboo scrimber in the real world.

## 1. Introduction

Currently, certain forms of bamboo materials like bamboo scrimber are extensively utilised in engineering works [1]. Bamboo scrimber has much better mechanical qualities and is more stable compared to raw bamboo and can therefore be used in a broader range of applications. As such, proper assessment and identification of its mechanical properties is vital before it can be practically applied [2,3].

In recent years, many studies have been carried out to achieve this aim. For example, Huang used different beam models to study the strength aspects of bamboo scrimber, determining its fundamental mechanical parameters [4,5]. Li performed tensile and compressive experiments on bamboo scrimber under various orientations (parallel and perpendicular to fibre) to study the mechanical constitutive relation of bamboo scrimber and produced four models that correctly define the stress–strain relation under given loading conditions [6]. Ma studied the high cycle fatigue of bamboo scrimber and determined the threshold value of its high cycle fatigue life. Moreover, its stress level has a great influence on the residual stiffness of specimens [7]. Shangguan analysed the effects of heat treatment on the strength of the material and discovered that it can lead to phenolic resin induration and can also change the crystallinity [8]. Yu investigated the strength of bamboo scrimber and found that it is mainly influenced by microstructural features and fibre matrix interface [9]. Song studied the fatigue life of bamboo scrimber by taking into account the contribution of both the fibre and the matrix and used a two-parameter Weibull distribution function to model the relationship between the load cycle and stress [10]. To investigate the effect of a specific heat treatment medium on the strength of bamboo scrimber, Yuan used hot oil, which adsorbed onto the fibre interface and resulted in a substantial decrease in strength. Moreover, it was found that the heat treatment duration must be long enough to exert an effect [11]. Cui conducted an experimental and theoretical study on the mechanical performance of bolt steel-to-laminated bamboo connections, based on which the bearing capacity of a component under given load conditions can be determined [12,13].

In earlier studies, bamboo scrimber was typically considered to be an anisotropic composite material; however, in recent years, increasing numbers of experts have found that the material usually exhibits obvious creep behaviour during the application process. Therefore, Liu examined the creep characteristics of laminated bamboo at various temperatures and found that the ratio of viscous creep to total deformation increased with temperature [14]. Liu also performed accelerated creep tests at different temperatures, thus extensively reducing the duration of the experiment and establishing a solid forecast of bamboo scrimber creep behaviour in the long-term [15]. In addition, the Burgers model was used by Wei and Chen to investigate different kinds of creep properties (tensile, compressive, bending); in this way, the detailed percentages of various strain components could be determined [16,17,18,19].

In a previous study, we analysed the tensile creep strain of different specimens under various stress levels [20]. The results showed that the model parameters varied significantly among specimens due to the inherent dispersive properties of the material, making detailed analysis difficult. Additionally, recent studies have indicated that the tensile and compressive mechanical properties of fibre-reinforced composite materials differ considerably [21], though whether the same model can adequately meet the requirements under compressive conditions remains unknown.

In this study, we conducted assessments of the compressive properties of bamboo scrimber. Firstly, four groups of experiments were carried out according to the given load. Then, several viscoelastic mechanical constitutive frameworks were used to decompose the progression of the time–strain compressive history of the material. The findings of this research provide a theoretical basis for informing safe and reliable design in engineering practice.

## 2. Materials and Methods

### 2.1. Material and Specimens

**Figure 1:**
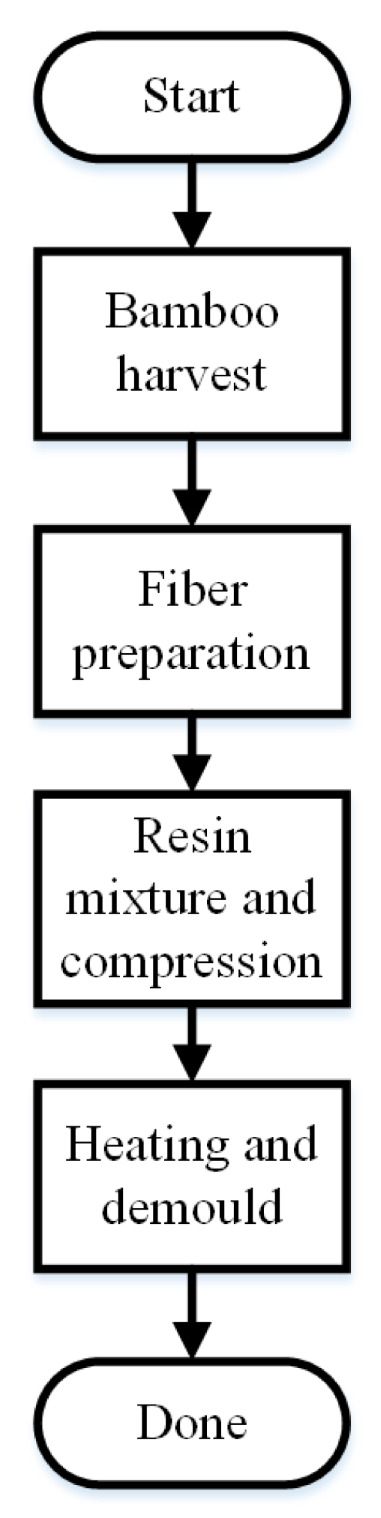
The manufacturing process of the bamboo scrimber (hot pressing).

In this paper, we aim to research the creep property of bamboo scrimber. For this composite material, the commonly used production method is hot pressing, which initially involves extracting natural bamboo fibres derived from raw bamboo using a chemical and rolling method. The fibres are then bundled together, stamped into predetermined steel boxes, and filled with phenolic resin. Lastly, the combination is kept at the required temperature (about 400 k) and pressure (about 20 MPa) in such a manner that the fibres are reglued. The raw material forming the sample in this work was moso bamboo (about 4 or 5 years old), and the sample was produced by the Tao Huajiang Company. In addition, the density of the recombinant bamboo was 1.08 g/cm^3^, the water content was 4.5%, and the glue content was 8.5%. The detailed manufacturing process is shown in Figure 1.

Figure 1 shows the detailed information of specimen manufacturing. The size was determined according to the ASTM standard 143-94 [22]. In this paper, 15 specimens were adopted, among which 11 were used for the compressive strength test and the other 4 were applied in the creep test.

### 2.2. Compressive Creep Experiment

**Figure 2:**
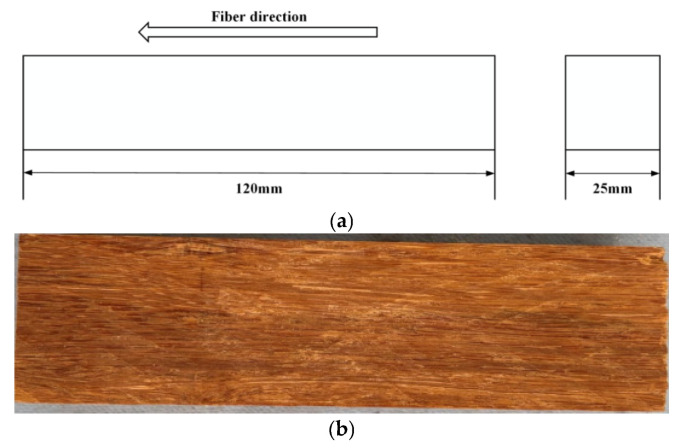
The main structural features of the test specimen in this paper. (**a**) the dimension parameters; (**b**) The real specimen.

The standard creep test is commonly used to research the creep performance of composite materials. The most frequently used parameter for evaluating this is the generated creep strain or displacement during the creep process. Figure 2 shows the corresponding test machine used to perform the creep test, from which the strain is recorded via an electronic extensometer fixed at the sample. According to a previous study, this approach is more suitable than the strain gaps which are applied in studying compressive creep performance [21]. The standard in this case is ASTM D2990-17 [23].

**Figure 3:**
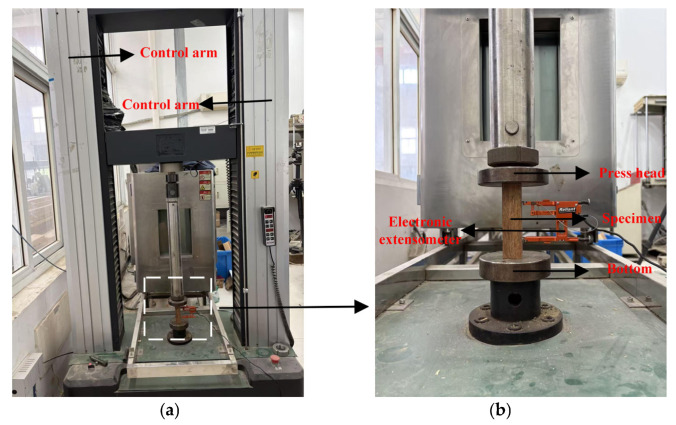
Compressive creep experimental equipment: (**a**) the overall equipment; (**b**) the partial area of the specimen.

Figure 3 shows the experiment equipment for the creep test. The sampling frequency during the test process was 1 min.

**Figure 4:**
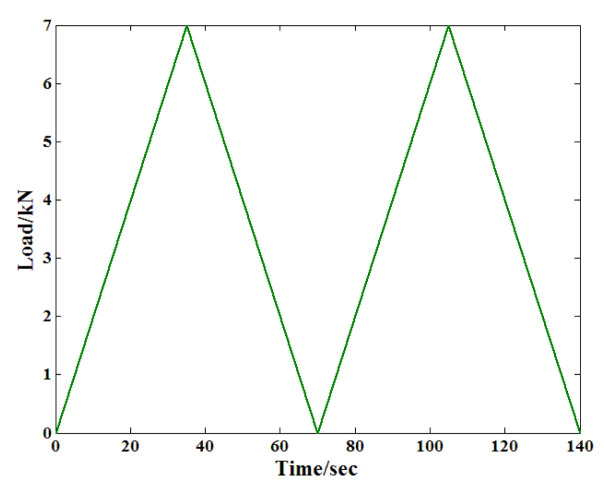
The pretreatment load before the creep test.

During this test, certain factors, such as the tiny gaps between the test equipment and the specimen, may result in measurement errors. As a result, corresponding preprocessing is necessary to guarantee the reliability of the recorded strain. Figure 4 shows the whole process of the load, from which it can be found that the load comprised two load cycles and lasted for 140 s. In addition, the load rate was generally steady throughout the process.

**Figure 5:**
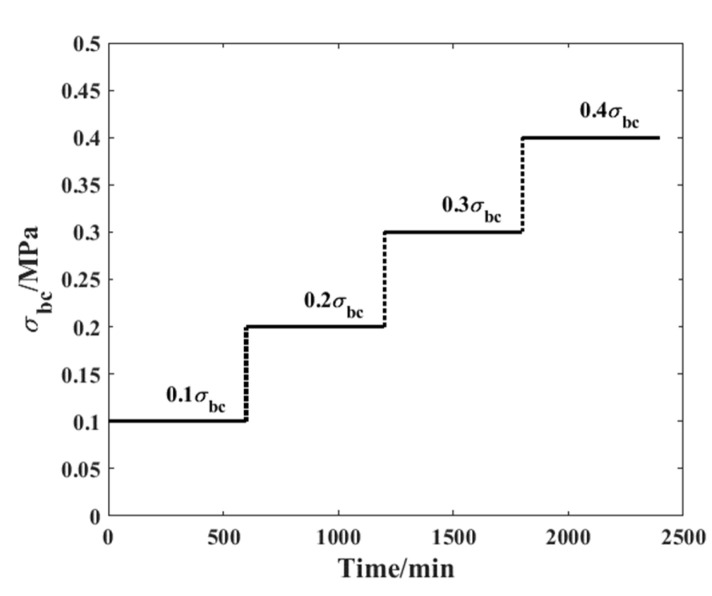
Load history applied in the compressive creep experiment.

In this study, a time-varying compressive load history was applied to the specimen to record the corresponding creep strain under different stress levels. Figure 5 demonstrates detailed information about the loading spectrum adopted in this paper, from which it can be found that the whole process comprised four time steps, each lasting 10 h. In addition, the load amplitude increased with the time step number and the material’s own compressive strength (from 10% to 40%). In this way, the compressive property of one specimen under various stress conditions can be studied.

### 2.3. Variable-Order Fractional Derivative Theory

**Figure 6:**
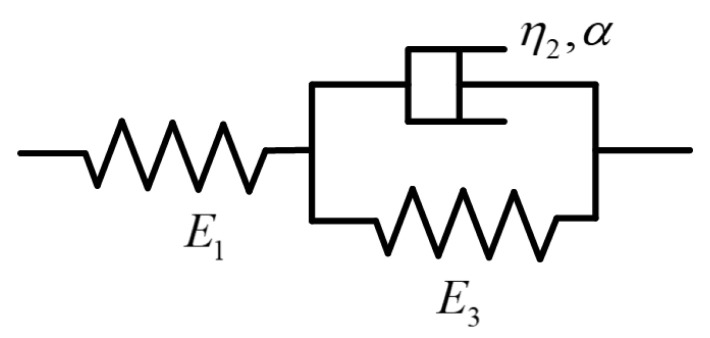
Schematic of the main components of the Kelvin-Voigt model.

The Kelvin-Voigt model was chosen to study the creep performance of the bamboo scrimber. Figure 6 shows the main components of the model, which consists of two spring bodies and one Koeller dashpot body [20]. One spring is connected in parallel with the Koeller dashpot, and this combined element is connected in series with the other spring. 

In previous studies, the Koeller dashpot has typically been regarded as the viscous component of a model. When a load is applied to the entire model, the corresponding stress–strain response can be expressed using the equations below:

(1) $$\sigma_{1}=\sigma_{2}+\sigma_{3}=\sigma_{0}$$

(2) $$\sigma_{1}\left(t\right)=E_{1}\varepsilon_{1}\left(t\right)$$

(3) $$\sigma_{3}\left(t\right)=E_{3}\varepsilon_{3}\left(t\right)$$

(4) $$\varepsilon_{3}\left(t\right)=\varepsilon_{2}\left(t\right)$$

where $\sigma_{0}$ denotes the total stress generated by the applied load; $\sigma_{1}$, $\sigma_{2}$, and $\sigma_{3}$ denote the stresses in the elastic spring body $E_{1}$, the Koeller dashpot body $\eta_{2}$, and the elastic spring body $E_{3}$, respectively; and $\varepsilon_{1}$, $\varepsilon_{2}$, and $\varepsilon_{3}$ denote the corresponding strains in these components. As indicated in Equations (3) and (4), the stress–strain relationships for the elastic spring bodies are well established; however, for the dashpot, there is no universally accepted definition for expressing the corresponding relationship. According to the conventional definition, this body is a typical fluid, the relationship with which can be expressed by the following equation:

(5) $$\sigma_{2}\left(t\right)=\eta_{2}\frac{d\varepsilon_{2}\left(t\right)}{dt}$$

where $\;\eta_{2}$ is the viscosity coefficient of the model. Consequently, the response strain under a given stress $\sigma_{0}$ can be expressed by the following three equations:

(6) $$\varepsilon_{1}\left(t\right)=\frac{\sigma_{0}}{E_{1}}$$

(7) $$\varepsilon_{2}\left(t\right)=\varepsilon_{3}\left(t\right)=\frac{\sigma_{0}}{E_{2}}(1-\exp(-\frac{E_{2}}{\eta_{2}}t))$$

(8) $$\varepsilon (t)=\varepsilon_{1}(t)+\varepsilon_{2}(t)=\varepsilon_{1}(t)+\varepsilon_{3}(t)=\frac{\sigma_{0}}{E_{1}}+\frac{\sigma_{0}}{E_{2}}(1-\exp(-\frac{E_{2}}{\eta_{2}}t))$$

In previous studies, the parameters of the Kelvin-Voigt model have typically been treated as material constants, unaffected by stress level or related conditions. In recent years, however, some researchers have found that the model may exhibit combined nonlinear and time-variant characteristics, with the fractional-order model considered an effective approach for nonlinear and time-variant analysis. The dashpot element defined under this theory can be expressed by the following equation [20]:

(9) $$\sigma_{2}=\eta_{2}\frac{d^{\alpha }\varepsilon_{2}\left(t\right)}{dt^{\alpha }}$$

where α is the order of the fractional derivative. Currently, several definitions of fractional derivatives have been proposed for addressing various theoretical or engineering problems. In this paper, the Caputo model was chosen. It is defined by the following equation [24,25]:

(10) $$D^{\alpha }f\left(t\right)=\frac{1}{\Gamma \left(1-\alpha \right)}\int_{0}^{t}\frac{f’\left(\tau \right)}{{\left(t-\tau \right)}^{\alpha }}d\tau$$

where Γ is the gamma function and τ is the intermediate variable during the derivative approach. As a result of this, the corresponding output of the dashpot body can be expressed as

(11) $$\begin{array}{cc} \sigma_{2} & =\eta \left(t\right)\frac{d^{\alpha }\varepsilon_{2}\left(t\right)}{dt^{\alpha }}\\ & =\eta \left(t\right)\int_{0}^{t}\frac{\varepsilon_{2}’\left(\tau \right)}{\Gamma \left(1-\alpha \right){\left(t-\tau \right)}^{\alpha }}d\tau \end{array}$$

As shown in Figure 5, the load history shows an obvious time-varying property. According to our previous research, when the fractional derivative is adopted to investigate the creep properties of composite materials, its order is significantly influenced by the applied stress level. Therefore, if the Caputo model is used to analyse the creep performance of bamboo scrimber, the order of the fractional derivative will also vary with the stress level. Based on this theoretical assumption, the stress–strain relationship of the dashpot body under this load can be expressed as

(12) $$\begin{array}{cc} \sigma_{2} & =\eta \left(t\right)\frac{d^{\alpha }\varepsilon_{2}\left(t\right)}{dt^{\alpha }}\\ & =\eta \left(t\right)\int_{0}^{t}\frac{\varepsilon_{2}’\left(\tau \right)}{\Gamma \left(1-\alpha \right){\left(t-\tau \right)}^{\alpha }}d\tau \\ & \approx \sum_{i=0}^{k}\eta \left(t_{i+1}\right)\int_{i\mu }^{\left(i+1\right)\mu }\frac{\partial \varepsilon_{2}\left(\tau \right)}{\partial \tau }\frac{1}{\Gamma \left[1-\alpha \left(t_{i+1}\right)\right]}\frac{d\tau }{{\left(t_{k+1}-\tau \right)}^{\alpha \left(t_{i+1}\right)}} \end{array}$$

As shown in Equation (6), $\eta \left(t_{i+1}\right)$ and $\alpha \left(t_{i+1}\right)$ are the viscosity coefficient and the fractional derivative order (*i* is the time step number), which can be approximatively considered as material constants within this range if the applied stress level remains unchanged. As shown in Equation (13), the time-varying effect of the variable-order Caputo model is governed by two factors: the current time and its variation with time. These factors result in a more pronounced time-varying effect compared with commonly used fractional derivative models, such as the Riemann–Liouville (R–L) model. Additionally, according to the defined load history, the duration of each stress level is identical (600 min); thus, based on this theory, the corresponding viscoelastic property of the dashpot body can be defined as

(13) $$\begin{array}{cc} \sigma_{2} & \approx \sum_{i=0}^{k}\eta \left(t_{i+1}\right)\frac{\varepsilon_{2}\left(t_{i+1}\right)-\varepsilon_{2}\left(t_{i+1}\right)}{\mu }\int_{i\mu }^{\left(i+1\right)\mu }\frac{1}{\Gamma \left[1-\alpha \left(t_{i+1}\right)\right]}\frac{d\tau }{{\left(t_{k+1}-\tau \right)}^{\alpha \left(t_{i+1}\right)}}\\ & =\sum_{i=0}^{k}\eta \left(t_{i+1}\right)\frac{\varepsilon_{2}\left(t_{i+1}\right)-\varepsilon_{2}\left(t_{i+1}\right)}{\mu }\int_{\left(k-i\right)\mu }^{\left(k-i+1\right)\mu }\frac{1}{\Gamma \left[1-\alpha \left(t_{i+1}\right)\right]}\frac{d\varphi }{\varphi^{\alpha \left(t_{i+1}\right)}}\\ & =\sum_{i=0}^{k}\eta \left(t_{i+1}\right)\frac{\varepsilon_{2}\left(t_{i+1}\right)-\varepsilon_{2}\left(t_{i+1}\right)}{\mu }\int_{i\mu }^{\left(i+1\right)\mu }\frac{1}{\Gamma \left[1-\alpha \left(t_{i+1}\right)\right]}\frac{d\varphi }{\varphi^{\alpha \left(t_{i+1}\right)}}\\ & =\sum_{i=0}^{k}\eta \left(t_{i+1}\right)\frac{\varepsilon_{2}\left(t_{i+1}\right)-\varepsilon_{2}\left(t_{i+1}\right)}{\mu }\frac{1}{\Gamma \left[1-\alpha \left(t_{i+1}\right)\right]}\int_{i\mu }^{\left(i+1\right)\mu }\frac{d\varphi }{\varphi^{\alpha \left(t_{i+1}\right)}}\\ & =\sum_{i=0}^{k}\eta \left(t_{i+1}\right)\left[\varepsilon_{2}\left(t_{i+1}\right)-\varepsilon_{2}\left(t_{i+1}\right)\right]\frac{\mu^{-\alpha \left(t_{i+1}\right)}}{\Gamma \left[2-\alpha \left(t_{i+1}\right)\right]}\left[{\left(i+1\right)}^{1-\alpha \left(t_{i+1}\right)}-i^{1-\alpha \left(t_{i+1}\right)}\right] \end{array}$$

As shown in Equation (14), the parameter *t* is the time quantum of the total loading spectrum and μ is the time quantum of each time step. The relationship between these factors can be expressed as

(14) t=(k+1)μ

According to this definition, it is possible to analyse the response strain of the dashpot body and the entire Kelvin-Voigt model once the stress level is given.

## 3. Results

### 3.1. Compressive Creep Results

**Figure 7:**
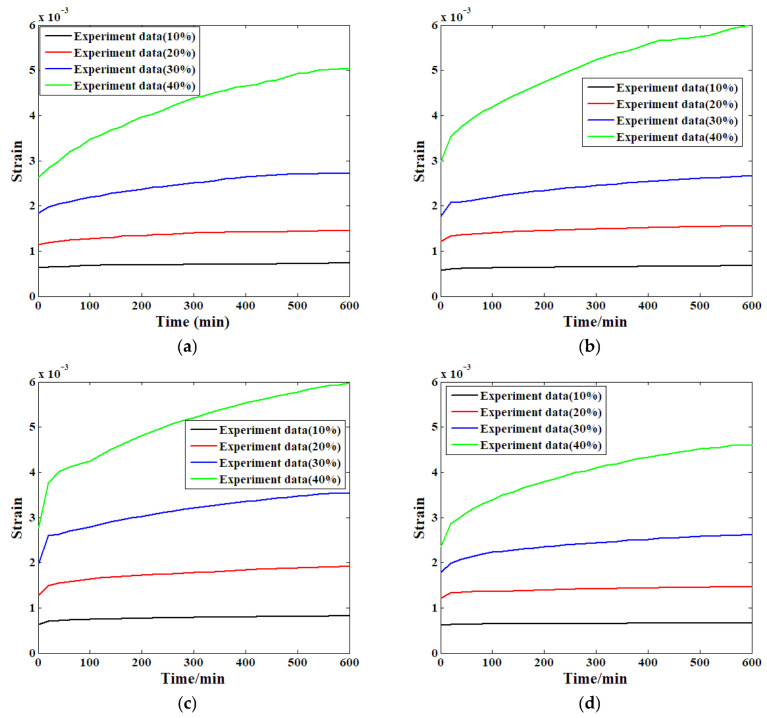
The creep test results of all specimens: (**a**) first; (**b**) second; (**c**) third; (**d**) fourth.

The compressive creep experiment was conducted according to the load history specified in Figure 5. Additionally, some factors that may influence creep behaviour, such as temperature and ambient humidity, were kept consistent throughout the whole process. This approach ensured that the influence of environmental conditions was accounted for in advance. In order to comprehensively verify the conclusion drawn from our research, four groups of creep tests were carried out to conduct a comparative study. Figure 7 shows the strain evolution process recorded during the creep experiment following the defined load history.

Figure 7a indicates the development of the first specimen’s creep strain at each of the four load stages. As can be seen, the curve with the lowest stress (10% of the compressive strength) slopes the least and the progression of strain is relatively constant. The strain curve gradient is steep at the larger stress level condition, and the strain development rate is observed to be high at the start of the evolution process compared to that at the lower stress level. Within about 100 min, the growth rate slows down and the curve slope becomes almost steady. The strain growth rate becomes notably large with the increase in stress level under the third loading condition. The minimum growth rate is observed at about 460 min in this stage of the experiment when the highest stress level is applied. The growth rate is highest (the relative increment is almost 90%) in the creep strain taken at the highest level of stress. It seems that the deceleration in the strain growth rate at this level of stress occurs at the end of the experimental stage. A comparison of the process of strain evolution of this specimen under each of the four levels of stress shows two main findings: namely, the more stress is introduced to the creep experiment, the larger the proportionality of the creep strain increase will be, and the deceleration point in the strain growth rate will be observed in the later test.

Figure 7b–d show all of the compressive creep test results from the other three specimens. Similarly to the first specimen, the increasing speed of the recorded creep strain is enhanced by the applied stress level, which may be attributed to the microstructural and structural characteristics of the bamboo scrimber. Based on the sample production method introduced previously, the recombinant bamboo is principally formed of bamboo fibres and phenolic resin, two materials wherein creep varies significantly. Generally speaking, a wide range of differences can exist between the rates of the increase and decrease in creep strain under the same level of stress and other mechanical property parameters, like the fact that their Youngs’ moduli are not identical, rendering the interface of the two materials vulnerable to shear stress due to the dissimilarity of the materials. In the initial creep test, both the fibre and matrix are capable of resisting creep deformation in the initial stage and their interstitial pores will not be damaged. The longer the experiment, the relatively higher the strain accumulated will be, leading to relatively higher displacement and destruction of the pores due to an inhomogeneity between the fibre and matrix. Consequently, this process recombined the sample into a new type; hence, the growth property of the strains was affected.

**Figure 8:**
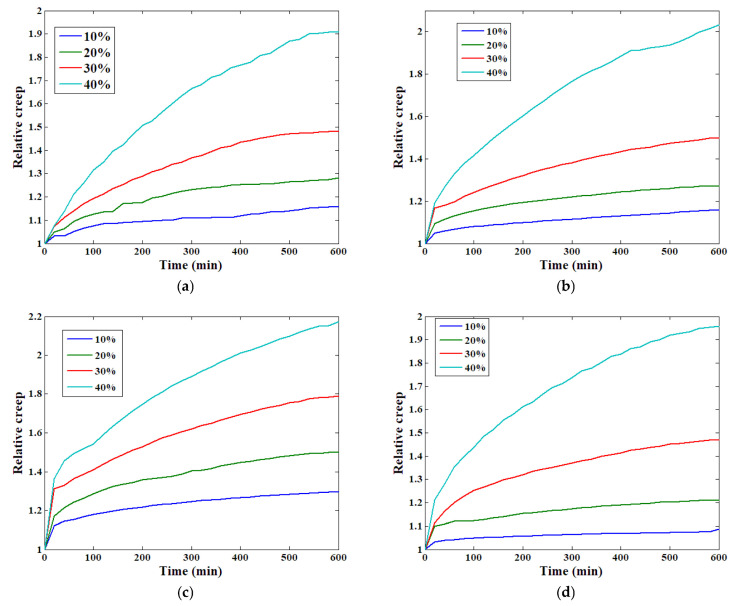
The relative creep of all specimens: (**a**) first; (**b**) second; (**c**) third; (**d**) fourth.

Relative creep was used to analyse the change in creep behaviour. The definition of this parameter, according to the ASTM standard D6815-09 [26], is

(15) $$\xi (t)=\frac{\varepsilon (t)}{\varepsilon (t=0)}$$

where ε(t) is the strain evolution process and ξ(t) is the relative creep. Based on this definition, the relative creep strain of all four specimens can be determined. Figure 8 shows the analysis of the relative creep, from which we find that the relative creep of the specimen is clearly influenced by the stress level applied on it. For all four specimens, the relative creep steadily increases with the stress level value. In other words, the material shows more obvious rheological properties with higher stress levels.

### 3.2. Creep Model Analysis Results

**Figure 9:**
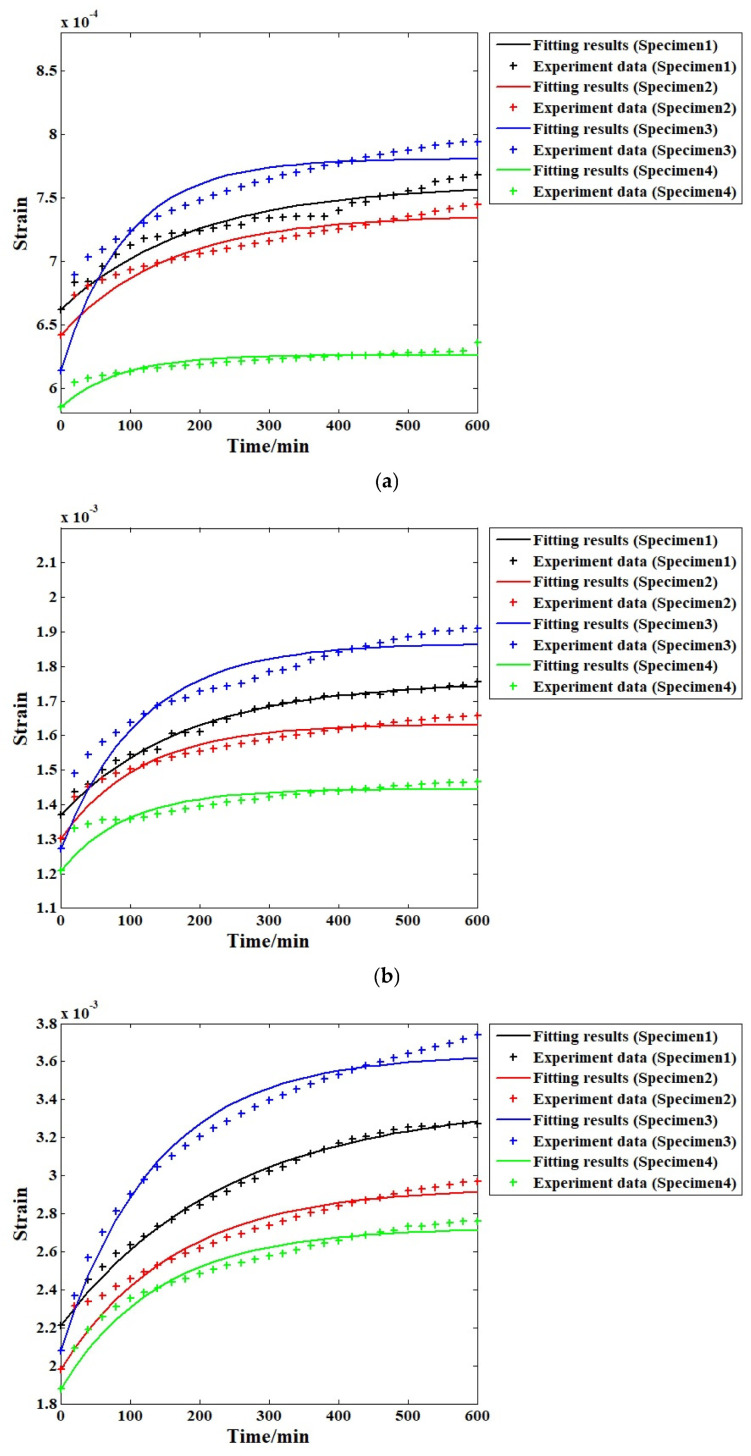

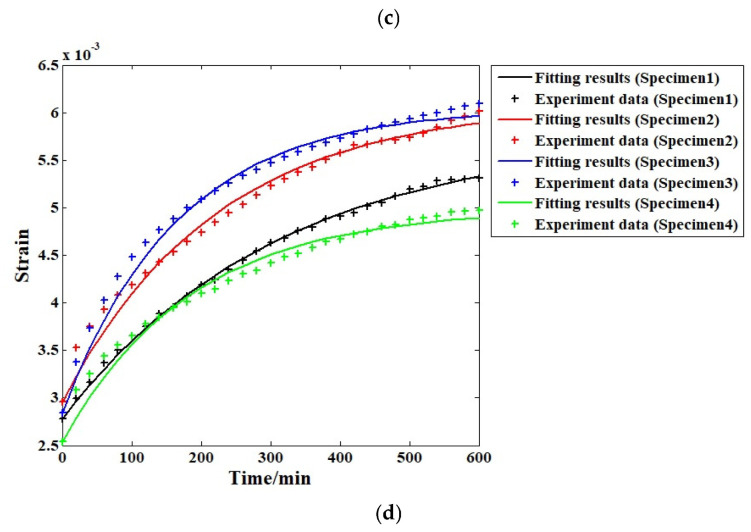
Fitting results of all cases using the conventional Kelvin-Voigt model under different stress levels: (**a**) 10%; (**b**) 20%; (**c**) 30%; (**d**) 40%.

In this paper, to ensure the comprehensiveness of the conclusions, both the conventional Kelvin-Voigt model and the VOF-derivative-based Kelvin-Voigt model were employed to fit the time–strain histories of the specimens under all four stress levels. Figure 9 presents the analysis results according to the conventional Kelvin-Voigt model. In addition, for all four specimens, the fitting results under the same stress level are combined into a single figure to provide further analysis of the stress level effect.

Figure 9a displays the results of the time–strain relationships of the four specimens under the first load stress (10% compressive strength), fitted using the conventional Kelvin-Voigt model. The result for the fourth specimen is relatively close to the experimental data, but those of the other three specimens are different compared with the experimental data. For the second stress level, as shown in Figure 9b, the fitting accuracy for the fourth specimen remains high, and the corresponding result for the first specimen shows marked improvement. However, for the other two specimens, the fitting results still differ significantly from the experimental data. For the third specimen, the difference is rather obvious to some degree. In Figure 9c, it is clear that when the 30% stress level is applied, higher fitting accuracy can be obtained for all four specimens compared to the second stress level. Under this condition, only the third specimen is unable to provide satisfactory results. The fitting results in Figure 9d indicate that under this highest stress level, the model can perform well when analysing the test results. Table 1 presents the detailed correlation coefficients for all of the cases under different stress levels, showing that higher applied stress levels lead to greater fitting accuracy of the conventional Kelvin-Voigt model, thereby representing the change in creep strain. At lower stress levels, the fitting results based on the traditional Kelvin-Voigt model usually exhibit substantial errors, making corresponding modifications and improvements necessary. This phenomenon can be explained by the definition of the traditional Kelvin-Voigt model itself. According to the relative creep analysis results shown in Figure 8, each specimen exhibits more obvious rheological behaviour when a higher stress level is applied, while for the traditional Kelvin-Voigt model, the dashpot body is also defined as a standard Newtonian fluid body, in accordance with the material’s properties under higher stress levels. This makes the model suitable for application in this situation.

On the other hand, another feature of the creep analysis results is that the creep behaviour of different specimens is also different even when the same stress level is applied, especially for the fourth specimen. This can be explained by the microstructural features of the material. According to a previous related study, in some degrees, the distribution of the fibre and phenolic resin within the specimen is not even, which results in the specimen’s mechanical properties usually including an obvious dispersion property, even though the raw material and manufacturing process are the same.

**Figure 10:**
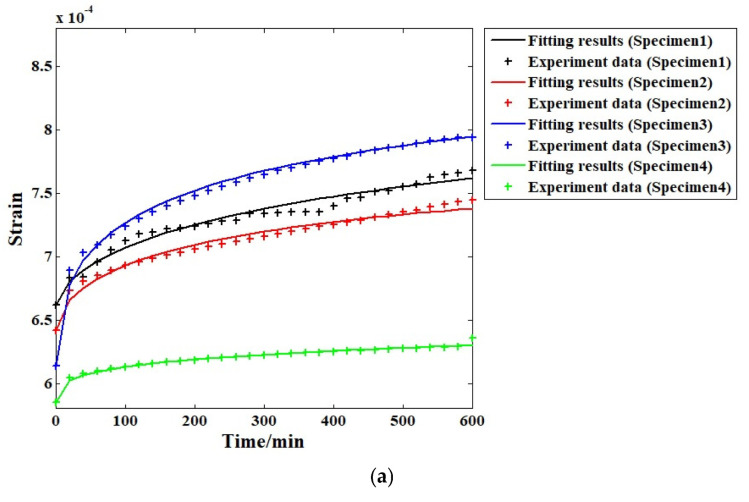

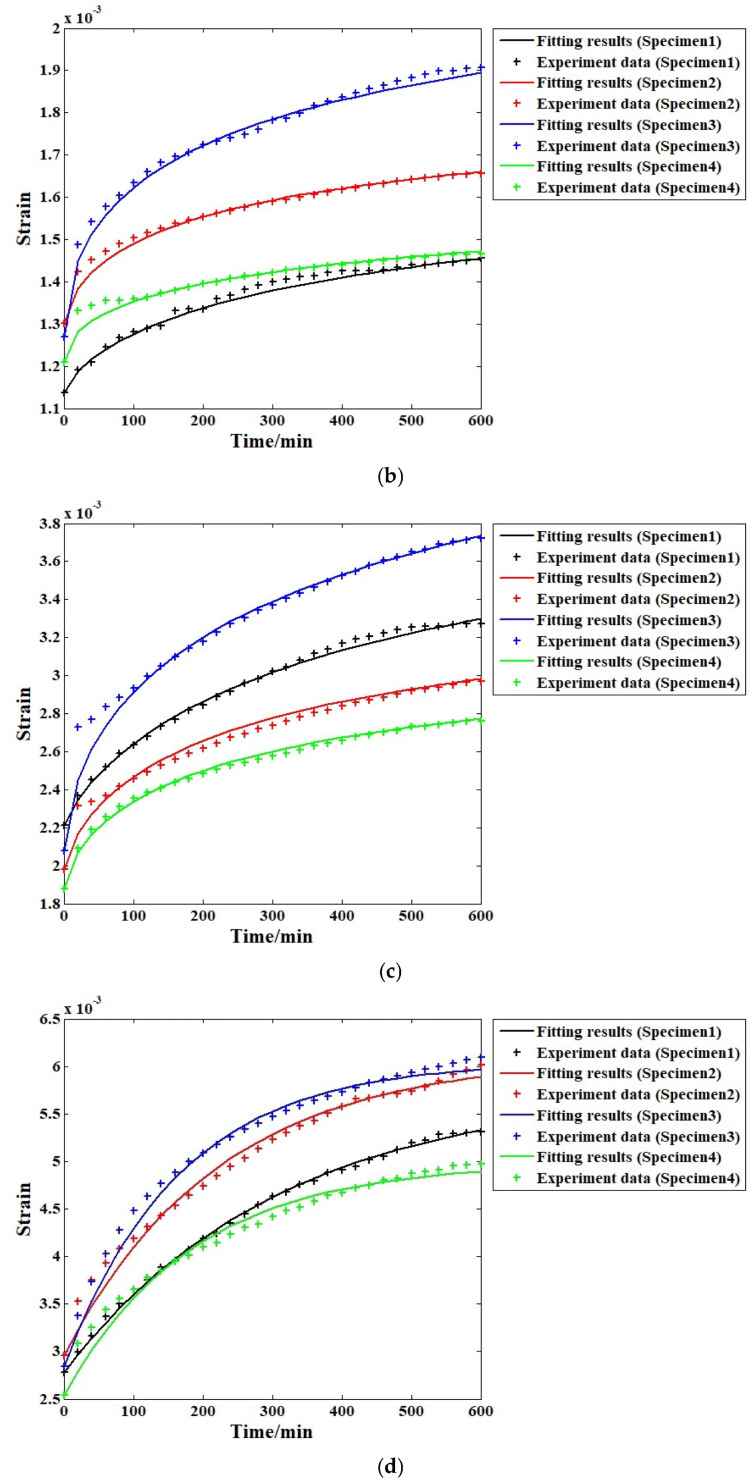
Fitting results of all cases using the VOF Caputo fractional derivative Kelvin-Voigt model under different stress levels: (**a**) first; (**b**) second; (**c**) third; (**d**) fourth.

The analysis results based on the proposed VOF model are shown in Figure 10 for comparison. Figure 10a–d show that the fitting results when the same level of stress is applied to the four specimens in the four variable-order Caputo fractional derivative Kelvin-Voigt model are significantly closer to the experimental data than the fitting results of the conventional Kelvin-Voigt model, particularly the third and fourth specimens. This is also witnessed in Figure 10b. In case of the second level of stress (20% compressive strength), the fitting outcomes in accordance with the suggested modified Kelvin-Voigt model are almost equal to the experimental results, proving that this model is clearly better than the ordinary one (Kelvin-Voigt model). The analysis results of the third level of stress are depicted in Figure 10c, where the fitting results of the proposed model are virtually equal to the results of the experiment. Figure 10d shows that when the maximum stress level is used, the fitting process is almost the same as that with the original experiment data.

Table 2 shows the detailed correlation coefficients for all stress levels and specimens based on the VOF Caputo fractional derivative Kelvin-Voigt model. Compared with the fitting results from the conventional model, the VOF Caputo fractional derivative Kelvin-Voigt model provides substantially higher accuracy in all cases (exceeding 98%), particularly at relatively lower stress levels. For higher stress levels, the accuracy remains sufficiently high as to meet engineering application requirements (exceeding 99%). Overall, this model is more suitable for compressive creep analysis in this field.

### 3.3. Model Parameter Analysis Results

**Figure 11:**
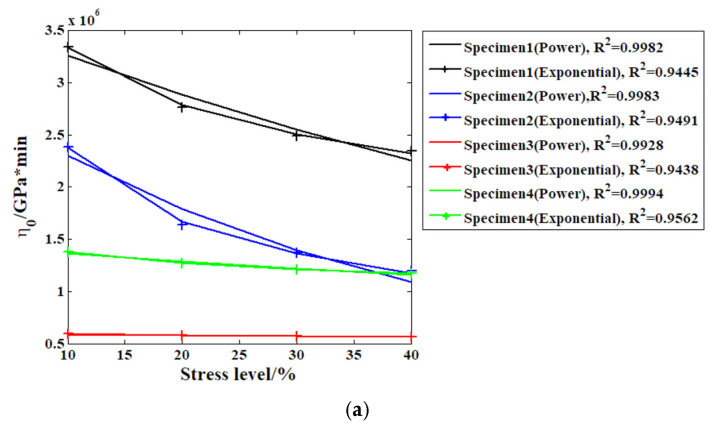

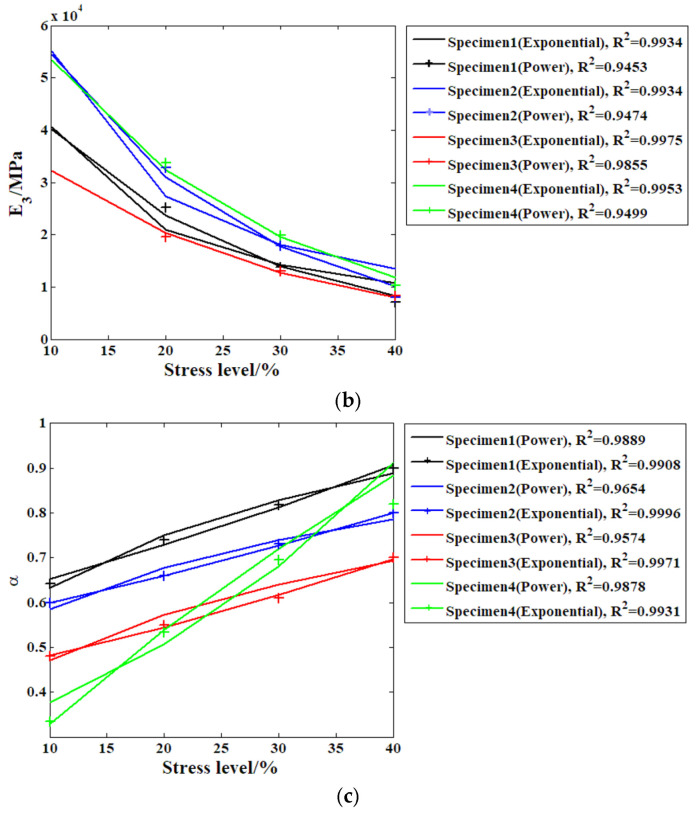
Stress level effect analysis results for different parameters: (**a**) viscosity coefficient $\;\eta_{2}$; (**b**) elasticity modulus *E*_3_; (**c**) order.

To continue to examine the applicability of the proposed model, we need to perform quantitative analysis of the fitting results. Previously, the effect of stress levels on the elastic properties of recombinant bamboo were studied in detail [21]. In this study, the effects of stress levels on the viscoelastic characteristics of a material are investigated, wherein the parallel-connected spring body and dashpot body are treated as viscoelastic properties based on the structural features of the Kelvin body itself. The order of the fractional derivative is another factor which may affect the viscoelastic properties of the Kelvin body. In this paper, two kinds of commonly used functions (the power and exponential) are adopted to analyse the stress level’s effect on these parameters; the results are represented in Figure 11.

As can be seen in Figure 11, the relationships between the three parameters of the Kelvin-Voigt model used in the study and the stress level have clear dissimilarities. In the case of the viscosity coefficient, as illustrated by Figure 11a, when the power function is introduced to graphically illustrate the relationship between the coefficient and the applied level of stress, the level of accuracy is satisfactory. All four correlation coefficients are above 0.99, which implies that this function is successful at capturing the power of stress levels on this model parameter. However, the results of fitting the same parameter with the aid of the exponential function demonstrate significantly lower accuracy; thus, the power function is more appropriate and applicable in this context compared to the exponential function. For the elastic modulus, the situation is different; the fitting results as per the exponential function are far more accurate than those associated with the power function and therefore the former is more suitable for use in this case. For the order of the fractional derivative, the exponential function also exhibits a far greater accuracy in establishing its correlation with the stress level applied as opposed to the power function. Using the developed relationships, the change in creep strain in samples subjected to different stress levels may be predicted, which is useful in achieving design requirements during fluctuating stress conditions. Moreover, among the model parameters of the different specimens, which were found based on their varying fitting outcomes, one can see a distinct difference in particular between the viscosity coefficient when the applied stress level is the same; there may be a more than four-to-five-times-greater relative difference. This is mostly the result of the scattering of the material properties. As per the bamboo scrimber production technique, this natural fibre-reinforced composite is produced by a pressing technique that might result in the uneven distribution of fibres and phenolic resin in a specimen and polytropy of mechanical characteristics [27].

## 4. Conclusions

In summary, the compressive creep performance of the bamboo scrimber was studied in detail. The results show that stress level has a strong impact on the compressive evolution course during the testing process, with the latter increasing relatively with the stress level. In addition, the proposed VOF-defined Kelvin-Voigt model can more accurately simulate the creep strain evolution process under all stress levels, and that the exponential function is a principal parameter that can successfully express the effect of the stress amplitude on the fractional derivative order of the Kelvin body modulus of elasticity. However, the power function was found to be more appropriate for describing the same effect on the coefficient of viscosity, the power type was found to be more appropriate in describing this relationship.

## Figures and Tables

**Table 1:** Correlation coefficients ($R^{2}$) based on the conventional Kelvin-Voigt model.

Specimen Number	10%	20%	30%	40%
1	0.8536	0.9917	0.9922	0.9984
2	0.8978	0.9178	0.9314	0.9822
3	0.883	0.9072	0.8923	0.9255
4	0.9213	0.8247	0.9545	0.9725
Mean	0.8889	0.9103	0.9426	0.9697

**Table 2:** Correlation coefficients ($R^{2}$) of the fitting results based on the VOF Caputo fractional derivative Kelvin-Voigt model.

Specimen Number	10%	20%	30%	40%
1	0.9842	0.9954	0.9981	0.9995
2	0.9888	0.9958	0.9918	0.9980
3	0.9969	0.9965	0.9957	0.9936
4	0.9922	0.9949	0.9984	0.9975
Mean	0.9905	0.9957	0.9961	0.9971

## Data Availability

The original contributions presented in this study are included in the article. Further inquiries can be directed to the corresponding author.
